# Energy Efficiency and Health Efficiency of Old and New EU Member States

**DOI:** 10.3389/fpubh.2020.00168

**Published:** 2020-06-09

**Authors:** Yongqi Feng, Xinye Yu, Yung-Ho Chiu, Tai-Yu Lin

**Affiliations:** ^1^School of Economics, Jilin University, Changchun, China; ^2^Department of Economics, Soochow University, Taipei, Taiwan; ^3^Department of Business Administration, National Cheng Kung University, Tainan City, Taiwan

**Keywords:** energy efficiency, health efficiency, old EU states, new EU states, meta-frontier dynamic network DEA

## Abstract

Environmental protection and health issues have always been of great concern. This study employed modified Meta-Frontier Dynamic Network Data Envelopment Analysis to explore the environmental pollution effects from energy consumption on the mortality of children and adults, tuberculosis rate, survival rate, and health expenditure efficiencies in 15 old EU states and 13 new EU states from 2010 to 2014. We calculated the overall efficiency scores and technology gap ratios for each old EU and new EU states as well as the efficiencies of non-renewable energy, renewable energy, PM2.5, CO_2_, labor, GDP, tuberculosis, child mortality, adult mortality, health expenditure efficiency, and survival efficiency at the health stage. The average annual overall efficiencies of the old EU states are higher than that of the new EU states. Whether in terms of energy efficiencies or health efficiencies, the inputs and outputs of the old EU states are always higher than that of the new EU states. Overall, developing countries in Eastern Europe are lagging behind in terms of energy and health efficiencies. At the same time, the efficiency of child mortality is lower than that of adult mortality, and the efficiency of PM2.5 is higher than that of CO_2_ in both old and new EU states.

## Introduction

The EU is a regional cooperative organization, and it has the highest degree of international economic integration in the world. However, the earlier European Community did not include environmental protection in its jurisdiction. Until the late 1960s, environmental policy was still considered as the internal affairs of each member country, which was customized and implemented by each member country. But by the end of 1970s, with the rapid economic growth of the European Union, environmental problems were deteriorating. In order to improve the economic development and living environment, the member states of the European Community incorporated environmental protection policies into the scope of joint management. In the course of its development, the EU has gradually improved its policies on environmental protection, increased its expenditure on environmental protection, and signed a number of important environmental agreements. These positive environmental policies play an important role in the sustainable economic development of EU countries.

At the 1998 Cardiff Summit, the heads of EU Member States made great efforts to promote the integration of environmental protection policies. At the summit, EU Member States first proposed a unified strategy to deal with climate change and formulated a common response model for all Member States to achieve the goals of the Kyoto Protocol. At the meeting, the EU also proposed that climate change policies must be implemented in parallel with energy policies. Subsequently, EU countries promoted the use of renewable energy and encouraged the use of new energy sources. At present, EU countries have implemented more than 35 measures to reduce carbon dioxide emissions, including a series of measures to limit carbon dioxide emissions from household cars and reduce carbon emissions from the industrial and transport sectors. The EU's energy-saving and emission reduction target is to reduce CO_2_ emissions by 20% by 2020 and to limit the increase in world temperature to within 2 degrees Celsius. At present, EU countries combine environmental policy with environmental technology to achieve their goals. The establishment of the ETS system is the unification of environmental protection policy and technology. ETS has provided more flexible carbon emission solutions for EU member countries, but because of the different economic development levels of EU member countries, ETS has met with opposition from many Eastern European countries. Even if Britain and Germany set up economic support funds to promote ETS development, many Eastern European countries are still worried about the hidden burden of carbon emissions.

The healthcare system can improve the level of economic development, and the achievements of the European Union regarding the healthcare system and health security are also remarkable. Among the European Union countries, the Netherlands have the most accomplished healthcare system; it has been ranked the first in Europe for 6 consecutive years. The investment in health care in the Netherlands accounts for more than 20% of the national budget and 40% of the local budget. Medical benefits in the Netherlands depend heavily on medical insurance, with the government subsidizing 60% of the cost of medical insurance. The medical system of EU countries occupies a large amount of economic resources, and the level of economic development of EU member countries thus also determines the extent of medical care.

However, the level of economic development of European countries varies. The per capita GDP of Western European countries is higher than the average level of the European Union, but the per capita GDP of Eastern European countries is much lower than the average level of the European Union. For example, in 2010, the EU's per capita GDP was $33,729, Germany and France's per capita GPD was $41,785 and $40,638, respectively, while Poland and Romania's per capita GDP in 2010 was only $12,599 and $8,209, respectively.

Does the imbalance of economic development affect the energy efficiency and environmental efficiency among EU member states? Is there some difference in energy efficiency and environmental efficiency between new and old member states? This problem is of great research value and worthy of critical thinking.

At present, many scholars have conducted a lot of research into energy and the environment. Among them, some scholars have used the DEA method to study energy efficiency. EU countries are also the research objects of energy and environment issues. At the same time, there are many studies into healthcare systems and health security in EU. As energy and environmental protection issues are closely related to our health problems, how to improve the efficiency of energy, environmental protection, and health input–output has become a problem worthy of study, particularly with focus on the case of differences that occur within in the development of European Union countries.

**Figure 1:**
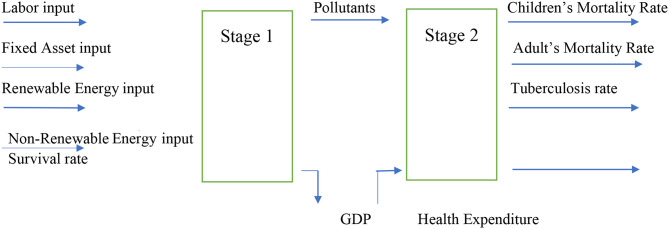
Process of input and output (data source: made by author).

There exists a input–output relationship—as well as an influence mechanism—between energy, environmental pollution, and health, as shown in Figure 1. When energy consumption and labor and capital input contribute to economic growth, they can result in environmental pollution, for example, carbon emissions and air pollution. The carbon emissions and air pollution have a very strong impact on respiratory, heart, and brain functions and can lead to some serious diseases; the government and society will have a significant fund for relational health expenditure for the health treatment.

Based on such an influence and transmission mechanism, this study employed modified meta-frontier dynamic network data envelopment analysis to explore the environmental pollution effects resulting from energy consumption on the mortality of children and adults, tuberculosis rate, survival rate, and health expenditure efficiencies in old EU and new EU states. This research has analyzed the energy and health efficiencies in old EU and new EU states. The first stage is the production stage, and we have investigated the energy efficiencies from this stage.

This study has two main contributions. First, energy, the environment, and health have been included in one model to comprehensively explore the energy and health efficiency of old EU countries and new EU states through a comparative analysis. Second, this study has divided energy into renewable energy and non-renewable energy, and it has also divided the mortality rate into children's mortality rate and adult's mortality rate.

The remainder of this article is organized such that the second section gives the literature review, the third section introduces the research model and method, the fourth section gives the empirical study results, and the fifth section presents the conclusions and implications.

## Literature Review

The issues of energy and health are widely discussed. The direction has mainly focused on environment and health. The first direction we have focused on has been the efficiency of energy and the environment. The second direction has emphasized the effects of pollution on human health.

On the aspects of energy and the environment, with particular focus on the economy, energy, pollution, and treatment therefor, Zhao et al. (1) have explored the relationship between air pollution and cycling. The results show that commuting trips are unlikely to be replaced by other models that might otherwise have improved the air quality of metropolitan areas such as Beijing. Lu et al. (2) used the dynamic slack-based data envelopment analysis (DEA) model to assess the environmental energy efficiency of high-income economies (including China) and explore the negative impacts on the environment to obtain a basis for energy-saving emission reduction methods or configurations by using 48 high-income economies (including China) from 2010 to 2014. Their empirical results show that economies with high energy efficiency have a large consumption of energy and are unable to reduce carbon dioxide emissions. Sueyoshi and Goto (3) used a non-oriented DEA model to explore the effect of environmental law on SO_2_ and NOx emission generated by United States coal-fired power plants. Liou and Wu (4) adopted the DEA model to assess CO_2_ emission control efficiency in developing countries. Choi et al. (5) explored the negative performance of CO_2_ efficiency in China by use of the SBM-DEA model. He et al. (6) showed that nitrogen oxides, SO_2_ (sulfur dioxide), and other polluting gases are damaging the environment and people's health, with a particular spike in incidences of many air pollution-related diseases in recent years. They used the modified undesirable dynamic two-stage DEA (data envelopment analysis) model to explore the economic, environmental, and health efficiencies of 30 provinces in China. Zhang and Choi (7) found the provincial environmental efficiency difference of China by SBM-DEA. Yang and Wang (8) explored that economic and CO_2_ emission efficiency required improvement in China during 2000–2007. According to Zhao et al. (9) CO_2_ and SO_2_ account for 40 and 60% of air pollution in China. The performance of power plants should be reformed. Yao et al. (10) used CO_2_ emission industrial data from China between 1998 and 2011 and the meta-frontier non-radial Malmquist performance index (MNMCPI) indicator to analyze CO_2_ emission efficiency. The results showed that the average CO_2_ emission in the east, central, and west declined in turn, but MNMCPI's efficiency (EC) rose. Wang et al. (11) used a non-oriented DEA model to evaluate the energy efficiency of China from 2008 to 2012, exploring how Shandong and Hainan performed well in natural management. The performance of western China was better than that of eastern and central China. Qin et al. (12) explored how the economic development of coastal areas in China was linked to energy performance, and they also explored Beijing and Hainan from 2000 to 2012. Saǧlam et al. (13) evaluated the energy efficiency of 39 US states by using a two-stage DEA model, and they found that many states exhibited good performance in CO_2_ emissions. Feng et al. (14) evaluated CO_2_ emissions efficiency in China and concluded that technical and management efficiency did not perform well. Cucchiella et al. (15) explored the low energy and environmental efficiency in the EU countries and energy consumption reduction was needed. Lu et al. (16) focused on sustainably and reasonably evaluating the characteristics and efficiency of the regional atmospheric environment; they calculated the atmospheric environmental efficiency and regional differences, which are based on the non-radial directional distance function DEA model. He et al. (17) focused the total factor energy efficiency index, established an epsilon-based measure-data envelopment analysis (EBM-DEA) model to measure the energy efficiency levels of 32 OECD countries during the period 1995−2016 when undesired outputs were included and not included. The effect of environmental factors on energy efficiency evaluation was compared by efficiency analysis and projection value analysis.

The research of European countries into the energy environment and health issues have drawn significant attention. Bampatsou et al. (18) evaluated the energy efficiency of 15 EU countries from 1980 to 2008. Nuclear energy negatively impacted countries. Bi et al. (19) showed the relationship between fuel consumption and thermal power regulation in China, exploring energy and environmental efficiency. Gomez-Calvet et al. (20) explored the poor energy efficiency of 25 EU countries through use of the distance direction function. Dumana and Kasman (21) researched the environmental efficiency of the EU from 1990 to 2011 by use of the parametric hyperbolic distance function. The original 15 countries in the EU could reduce CO_2_ emission while energy consumption was reduced. Cecchini et al. (22) used the DEA model to explore the energy efficiency of the European livestock industry. The results showed that the improvement of European livestock technology exhibited was significantly related to the reductopm pf carbon dioxide emissions. Cecchini et al. (22) accessed the energy efficiency of the European livestock industry and found that technology played an important part in CO_2_ reduction.

Many studies have been carried out into the effect of environmental pollution on human health. Chai et al. (23) examined the efficiency of China's health system to better understand the underlying causes of the variation in efficiency across provinces. By using a bootstrapping data envelopment technique, they focused on the performance in maternal health, child health, and non-communicable diseases (NCDs) in the 31 provinces of mainland China during 2015. Nansai et al. (24) quantified the mortality and economic loss in individual Asian countries caused by the PM2.5 emissions induced by the consumption of the world's five highest-consuming countries (the US, China, Japan, Germany, and the UK). The result shows that in 2010 alone, the economic impact of these five countries' consumption caused a loss of almost 45 billion US dollars due to the premature deaths of more than 1 million people in Asia, including 15,000 children younger than 5 years old. Wang (25) studied the impact of pollutant emissions in China on population health. An increase in PM10 and SO_2_ damaged population health. Fischer et al. (26) explored how long-term exposure to PM10 and NO_2_ increased mortality in the Netherlands. Yang et al. (27) explored how pollutants had a significant impact on the health of women and the elderly in 24,845 adults (aged 18–74 years) in three cities in China in 2009. Li et al. (28) found that health problems were caused by PM2.5 emissions and economic losses in Beijing, and energy consumption and PM2.5 emissions increased mortality. Liu et al. (29) adopted the LEAP (Long-Range Energy Alternative Planning System) model to discuss the impact of CO_2_ emissions and health problems. Acute bronchitis was related to PM10. Dauchet et al. (30) explored the impact of short-term exposure to air pollution on lung function in northern French cities. Short-term exposure to air pollution had a negative effect on lung health. Carlton et al. (31) evaluated the link between the average air exchange rate (AAER) and the respiratory function of low-income urban households using a structured questionnaire from a standard instrument to estimate the annual data for each family. Households with higher AAERs suffered from chronic coughing, asthma, and asthma-like symptoms. Shen et al. (32) evaluated health risks by the use of the Air Quality Index (AQI) and the Health Risk Air Quality Index (HAQI) and found that AQI was underestimated. Ljungmau et al. (33) explored the effect of long-term and short-term air pollution exposure on arterial stiffness. Long-term exposure to PM2.5 had no effect on arterial stiffness. Torres et al. (34) researched SO_2_ exposure and health effects; they found that air pollutants increased in Alentejo and Lisbon. Chen et al. (35) explored how PM2.5 and PM10 damaged the lung function of primary school children. Knibbs et al. (36) researched the impact of NO_2_ on children aged 7–11 in 12 cities in Australia. Among 2,630 children, the rate of asthma was 14.9% related to outdoor NO_2_. Roberts et al. (37) explored the relationship between air pollutants and mental health in childhood. The result showed that air pollution did not significantly affect mental health. Zaman et al. (38) analyzed the relationship between energy consumption, the environment, health, and economic growth in BRICS countries (Brazil, Russia, India, China, and South Africa) in 1975. Environmental variables affected growth. Health expenditures and infrastructure were related to mortality in BRICS countries. Bai et al. (39) provided a health cost accounting method for China and provided suggestions. Chen et al. (35) investigated the relationship between air pollutants and children's health in China from 2014 to 2016. The results showed that higher air pollution exposure is associated with an increased prevalence of respiratory disease in young children. Fioravanti et al. (40) studied whether air pollution and vehicle traffic exposure in 2003–2004 affected obesity among children aged 4 and 8 years in Rome. The results showed that there was no linkage between vehicle and child overweight/obesity. Huang et al. (41) analyzed the relationship between Beijing residents and the social economy. The results showed that household income and education were impacted by air pollutants. Kasdagli et al. (42) explored the effect of air pollution on Parkinson's disease (PD). The results showed that PM2.5 and PM10 had effect on PD. Wilkinson et al. (43) explored low-income countries. They found that the dissemination of affordable technology remains a vital priority to alleviate the burdens of indoor air pollution and other health effects in individuals obliged to rely on biomass fuels for cooking, heating, as well as the improvement of access to electricity, which would have many benefits to health.

## Research Method

### The Modified Meta Dynamic Network Model

As this study considered both undesirable outputs and regional differences, we can modify Tone and Tsutsui's (44) dynamic network model and O'Donnell et al.'s (45) meta-frontier model to be the modified meta-frontier dynamic network model. Therefore, the modified meta-frontier dynamic network model is as explained below.

Suppose there are *n* number of *DMUs* (*j* = 1, …, *n*), each having *k*divisions (*k* = 1, …, *K*) and *T*time periods (*t* = 1, …, *T*). Each of the *DMUs* have an input and output at time period *t* and a carryover (link) to the next *t*+1 time period.

Set *m*_*k*_ and *r*_*k*_ to represent the input and output in each division *K*, with (*k, h*)*i* representing divisions *k* to *h*; $L_{hk}^{\;}$ is the *k* and *h* division set, and the input, output, links, and carryover definitions are outlined in the following.

**Inputs and outputs**

$X_{ijk}^{t}\in R_{+}$ $\left(i=1,\ldots ,m_{k}^{\;};j=1,\ldots ,n;K=1\ldots ,K;t=1,\ldots ,T\right)$ refers to input *i*at time period *t*for *DMU*_*j*_ division *k*.
$y_{rjk}^{t}\in R_{+}$ $\left(r=1,\ldots ,r_{k}^{\;};j=1,\ldots ,n;K=1\ldots ,K;t=1,\ldots ,T\right)$ refers to output r in time period t for *DMU*_*j*_ division *k*; if part of the output is not ideal, it is considered an input for the division.

**Links**

$Z_{j\left(kh\right)t}^{t}\in R_{+}$ $\left(j=1;\ldots ;n;l=1;..;L_{hk}^{\;};t=1;\ldots ;T\right)\;0$ refers to the period t links from *DMU*_*j*_ division *k* to division *h*, with $L_{hk}^{\;}$ being the number of *k* to *h* links.
${\text{Z}}_{\text{j}\left(\text{kh}\right)\text{t}}^{\text{t}}\epsilon$ R_+_(j =1;…; n; l = 1;…; L_kh_; t = 1;…; T).

**Carryovers**

$Z_{jkl}^{\left(t,t+1\right)}\in R_{+}$ $\left(j=1,\ldots ,n;l=1,..,L_{k}^{\;};k=1,\ldots k,t=1,\ldots ,T-1\right)$ refers to the carryover of *t* to the *t*+1 period from *DMU*_*j*_ division *k* to division *h*, with $L_{k}^{\;}$ being the number of carryover items in division *k*.

**Meta-frontier (MF)**

With different management, environments, and resources, all firms (N) are composed of g groups of DMU (N = N_1_ + N_2_ +….+ N_G_; x_ij_ and y_rj_ are jth DMU's (j = 1, 2, …, N) input i (i = 1, 2, …, m) and final good r (r = 1, 2, …, s). Under the meta-frontier, decision-making unit k can choose the final output weight that maximizes its value, and the efficiency of decision-making unit k can thus be solved by the following linear programming procedure.

(1) $$\begin{array}{l} \rho^{*}=\min\frac{\frac{1}{T}\sum_{t=1}^{T}W^{t}\left[1-\frac{1}{m+ninput}\left[\sum_{g=1}^{G}\sum_{i=1}^{m}\frac{S_{it}^{-}}{X_{iot}}+\sum_{g=1}^{G}\sum_{r=1}^{ninput}\frac{S_{rt}^{input}}{Z_{rot}^{input}}\right]\right]}{\frac{1}{T}\sum_{t=1}^{T}W^{t}\left[1+\frac{1}{S_{1}+S_{2}}\left[\sum_{g=1}^{G}\sum_{l=1}^{S1}\frac{S_{jt}^{+g}}{y_{lot}^{g}}+\sum_{g=1}^{G}\sum_{l=1}^{S2}\frac{S_{jt}^{-b}}{y_{lob}^{b}}\right]\right]}\\ \;s.t.\;\sum_{g=1}^{G}\sum_{\partial =1}^{n}Z_{ijtg}\lambda_{jg}^{t}\sum_{g=1}^{G}\sum_{\partial =1}^{n}Z_{ijtg}\lambda_{jg}^{t+1}\;\left(vi|t=1\ldots i-1\right) \end{array}$$

(1) is the linkage of period t and t+1.

(2) $$\begin{array}{l} X_{iot}=\sum_{g=1}^{G}\sum_{\partial =1}^{n}X_{ijtg}\lambda_{jg}^{t}+S_{it}\;\left(i=1\cdots m,t=1\cdots i\right)\\ \;y_{lot}=\sum_{g=1}^{G}\sum_{l=1}^{s1}y_{lot}^{+g}\lambda_{j}^{t}-s_{lt}^{+g}\left(l=1,\ldots ,s1;t=1,\ldots ,T\;\right)\\ \;y_{lot}=\sum_{g=1}^{G}\sum_{l=1}^{s2}y_{lot}^{-b}\lambda_{j}^{t}-s_{lt}^{+b}\;\left(l=1,\ldots ,s2;t=1,\ldots ,T\right)\\ Z_{iot}^{good}=\sum_{g=1}^{G}\sum_{\partial =1}^{n}Z_{ijtg}^{good}\lambda_{jg}^{t}-S_{it}^{t}\;\left(i=1\cdots ngood;t=1\cdots i\right)\\ \;\sum_{g=1}^{G}\sum_{\partial =1}^{n}\lambda_{jg}^{t}=1\left(t=1\cdots i\right)\\ \;\lambda_{jg}^{t}\geq 0,S_{it}^{-}\geq 0,S_{it}^{+}\geq 0,S_{it}^{good}\geq 0 \end{array}$$

Therefore, with Equation (2), we can find the overall technical efficiency (OTE) value of all DMUs under the meta-frontier model.

**Dynamic meta-frontier model**

Suppose the manufacturer is divided into g group decision-making units, and the DMU under each group boundary will choose the most favorable final output weight. Therefore, the efficiency of the DMU at the group frontier will be solved by the following equation

(3) $$\theta_{0}^{*}=\min\frac{\frac{1}{T}\sum_{t=1}^{T}W^{t}\left[1-\frac{1}{m+ninput}\left[\sum_{i=1}^{m}\frac{S_{it}^{-}}{X_{iot}}+\sum_{r=1}^{ninput}\frac{S_{rt}^{input}}{Z_{rot}^{input}}\right]\right]}{\frac{1}{T}\sum_{t=1}^{T}W^{t}\left[1+\frac{1}{S_{1}+S_{2}}\left[\sum_{l=1}^{S1}\frac{S_{jt}^{+g}}{y_{lot}^{g}}+\sum_{l=1}^{S2}\frac{S_{jt}^{-b}}{y_{lob}^{b}}\right]\right]}$$

$$\begin{array}{l} St\sum_{j=1}^{n}z_{ijt}^{\alpha }\lambda_{j}^{t}=\sum_{j=1}^{n}z_{ijt}^{\alpha }\lambda_{j}^{t+1}\left(\forall i;t=1,\ldots ,T-1\right)\\ \;x_{iot}=\sum_{j=1}^{n}x_{ijt}\lambda_{j}^{t}+s_{it}^{-}\;\left(i=1,\ldots ,m;t=1,\ldots ,T\;\right)\\ \;y_{lot}=\sum_{l=1}^{s1}y_{lot}^{+g}\lambda_{j}^{t}-s_{lt}^{+g}\;\left(l=1,\ldots ,s1;t=1,\ldots ,T\right)\\ \;y_{lot}=\sum_{l=1}^{s2}y_{lot}^{-b}\lambda_{j}^{t}-s_{lt}^{-b}\;\left(l=1,\ldots ,s2;t=1,\ldots ,T\right)\\ \;z_{iot}^{good}=\sum_{j=1}^{n}z_{iot}^{good}\lambda_{j}^{t}-s_{it}^{good}\;\left(i=1,\ldots ,ngood;t=1,\ldots ,T\right) \end{array}$$

$$\begin{array}{l} \sum_{j=1}^{n}\lambda_{j}^{t}\;=1\;\left(t=1,\ldots ,T\right)\\ \lambda_{j}^{t}\geq 0,s_{it}^{-}\geq 0,\;s_{it}^{+}\geq 0,s_{it}^{good}\geq 0 \end{array}$$

**Technology gap ratio (TGR)**Since the meta-frontier model contains g groups, the technical efficiency of the meta-frontier (MFE) will be less than the technical efficiency of the group frontier (GFE). The ratio value, called the technology gap ratio (TGR), is shown as,

(4) $$\begin{array}{r} TGR=\frac{MFE}{GFE} \end{array}$$

### The Efficiency of Input and Output

**Figure 2:**
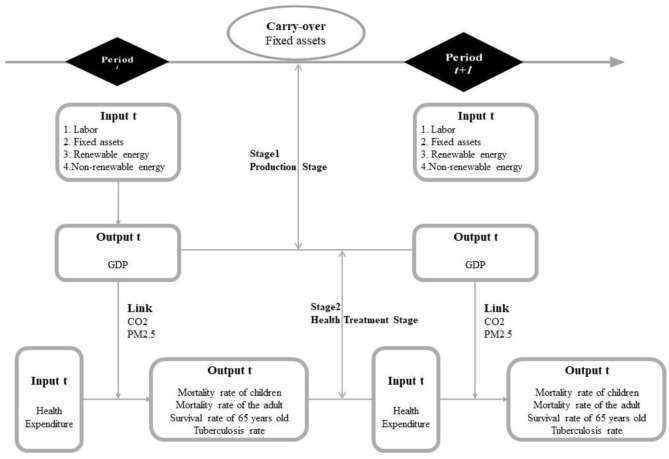
Two-stage meta dynamic network DEA model (Data source: made by author).

We followed Hu and Wang's (46) total-factor energy efficiency index to overcome any possible bias in the traditional energy efficiency indicator. There are 11 key features of this present study: Labor efficiency, non-renewable energy efficiency; renewable energy efficiency, GDP efficiency, Health Expenditure efficiency, Tuberculosis rate efficiency, Mortality rate of children efficiency, Mortality rate of adult efficiency, 65-years-old survival rate, CO_2_ efficiency, and PM2.5 efficiency. In our study, “I” represents area, and “t” represents time. More detail is in Figure 2. The 11 efficiency models are defined in the following section.

First stage: Production Efficiencies

$$\begin{array}{l} \text{Labor efficiency}=\frac{\text{Target Labor input }(\text{i},\text{ t})}{\text{Actual Labor input }(\text{i},\text{ t})}\\ \text{Non-renewable Energy efficiency}\\ \;=\frac{\text{Target non-renewable energy input }(\text{i},\text{ t})}{\text{Actual non-renewable energy input }(\text{i},\text{ t})} \end{array}$$

$$\begin{array}{l} \text{Renewable Energy efficiency}=\frac{\text{Target renewable energy input }(\text{i},\text{ t})}{\text{Actual renewable energy input }(\text{i},\text{ t})}\\ \text{ GDP efficiency=}\frac{\text{Actual GDP desirable output }(\text{i},\text{ t})}{\text{Target GDP desirable output }(\text{i},\text{ t})}\\ \;{\text{CO}}_{2}\text{ efficiency=}\frac{\text{Target }{\text{Co}}_{\text{2}}\text{ Undesirable output }(\text{i},\text{ t})}{\text{Actual }{\text{Co}}_{\text{2}}\text{ Undesirable output }(\text{i},\text{ t})}\\ \text{ PM2}.\text{5 efficiency=}\frac{\text{Target Pm2}.\text{5 Undesirable output }(\text{i},\text{ t})}{\text{Actual Pm2}.\text{5 Undesirable output }(\text{i},\text{ t})} \end{array}$$

Second stage: Health Efficiencies

$$\begin{array}{l} \text{ Health Expenditure efficiency}\\ \;=\frac{\text{Target Health Expenditure input }(\text{i},\text{ t})}{\text{Actual Health Expenditure input }(\text{i},\text{ t})}\\ \text{Tuberculosis rate efficiency}\\ \;=\frac{\text{Target Tuberculosis rate output }(\text{i},\text{ t})}{\text{Actual Tuberculosis rate output }(\text{i},\text{ t})}\\ \text{ Mortality rate of children efficiency}\\ \;=\frac{\text{Target Mortality rate of children output }(\text{i},\text{ t})}{\text{Actual Mortality rate of children output }(\text{i},\text{ t})}\\ \text{ Mortality rate of adult efficiency}\\ \;=\frac{\text{Target Mortality rate of adult output }(\text{i},\text{ t})}{\text{Actual Mortality rate of adult output }(\text{i},\text{ t})}\\ \text{ Survival rate of 65-years-old efficiency}\\ \;=\frac{\text{Actual Survival rate of 65-years-old desirable output }(\text{i},\text{ t})}{\text{Target Survival rate of 65-years-old desirable output }(\text{i},\text{ t})} \end{array}$$

In the first stage, if the target labor, non-renewable energy efficiency, and renewable energy efficiency input equals the actual input, then the labor, non-renewable energy efficiency, and renewable energy efficiency equals 1, indicating overall efficiency. If the target labor, non-renewable energy efficiency, and renewable energy efficiency input is less than the actual input, then the labor, non-renewable energy efficiency and renewable energy efficiency are <1, indicating overall inefficiency. If the target GDP desirable output is equal to the actual GDP desirable output, then the GDP efficiency equals 1, indicating overall efficiency. If the actual GDP desirable output is less than the target GDP desirable output, then the GDP efficiency is <1, indicating overall inefficiency. If the target the CO_2_ and PM2.5 undesirable outputs equal the actual undesirable outputs, then CO_2_ and PM2.5 efficiencies equal 1, indicating overall efficiency. If the target CO_2_, and AQI undesirable outputs are less than the actual undesirable outputs, then CO_2_ and PM2.5 efficiencies are <1, indicating overall inefficiency.

In the second stage, if the target Health Expenditure input equals the actual input, then the Health Expenditure efficiencies equal 1, indicating overall efficiency. If the target Health Expenditure input is less than the actual input, then the Health Expenditure efficiencies are <1, indicating overall inefficiency. If the target Survival rate of 65-years-old desirable output is equal to the actual Survival rate of 65-years-old desirable output, then the Survival rate of 65-years-old efficiency equals 1, indicating overall efficiency. If the 65-years-old survival rate's desirable output is less than the target 65-years-old survival rate's desirable output, then the 65-years-old survival rate's efficiency is <1, indicating overall inefficiency. If the target Tuberculosis rate efficiency, Mortality rate of children efficiency, and Mortality rate of adult efficiency undesirable output equal the actual undesirable output, then the Tuberculosis rate efficiency, Mortality rate of children efficiency, and Mortality rate of adult efficiency equal 1, indicating overall efficiency. If the target the Tuberculosis rate efficiency, Mortality rate of children efficiency, and Mortality rate of adult efficiency undesirable outputs are less than the actual undesirable outputs, then the Tuberculosis rate efficiency, Mortality rate of children efficiency, and Mortality rate of adult efficiency are <1, indicating overall inefficiency.

## Empirical Study

### Data Sources and Description

This study compares the energy efficiency and health efficiency in old and new EU states from 2010 to 2014. The total data are extracted from World Development Indicators of the World Bank (47). There are 15 old EU states that joined the EU before 1996. We choose 13 states as new EU states—states that joined the EU from 2004 until the present date (More detail is in Table 1).

**Table 1:** Input and output variables.

**Stage**	**Input variables**	**Output variables**	**Link**	**Carry over**
Stage 1	Labor by million persons	GDP by billion dollars	CO_2_ by million ton	Fixed assets by billion dollars
	Renewable energy by Mega Joule		PM2.5 by micrograms per cubic meter	
	Non-renewable energy by Mega Joule			
Stage 2	Health Expenditure by billion dollars	Mortality rate of children (<5 years old) by percent		
		Mortality rate of adults (from 15 to 65-years-old) by percent		
		Survival rate of 65-years-old by percent		
		Tuberculosis rate by %		

*Data Source: World Development Indicators of the World Bank, https://data.worldbank.org.cn/indicator/*.

#### The First Stage: Production Stage

The input variables were such that labor meant the numbers of employees in each country by the end of each year, and the unit was measured in millions of people. We used the number of people over 15 years of age multiplied by the proportion of people over 15 years of age who are employed to calculate the number of people who are employed.

Renewable energy was measured as renewable energy consumption in each country every year, and the unit was mega joules. The data of renewable energy are the share of renewable energy in total final energy consumption.

Non-renewable energy was measured by non-renewable energy consumption in each country each year, and the unit was mega joules. The data of non-renewable energy are the share of fossil energy in total final energy consumption.

Output variables were such that GDP (desirable output) was measured in the GDP of each country each year, and the unit was in billions dollars at the current price.

#### The Second Stage: The Health Treatment Stage

Input variables were such that healthy expenditure was measured in the total annual health expenditure in each country, and the unit was in billions of dollars.

Output variables were such that the mortality rate of children (undesirable output) was defined by data from children <5 years of age in each country each year, and the unit was in percent.

The mortality rate of the adults (undesirable output) was measured in the mortality rate of adults <65 years of age, but more than 5 years of age, in each country each year, and the unit was in percent. The mortality rate of the aged data are female adult mortality plus male adult mortality.

The survival rate (desirable output) was measured in the survival rate of those over 65 years of age in each country each year, and the unit was in percent.

The tuberculosis rate (undesirable output) was measured in the effect of excessive content of CO_2_ and PM2.5 in the air on the reduction of people's immunity to pulmonary tuberculosis, which could thus increase the incidence of pulmonary tuberculosis. The tuberculosis rate was measured as the rate in each country each year, and the unit was in percent.

#### Link Production Stage and the Health Treatment Stage Variables

CO_2_ is the CO_2_ emissions in each country each year. The unit used was in millions of ton.

PM2.5 is the content of PM2.5 in the air in each country each year. The unit used was micrograms per cubic meter.

#### Carry Over Production Stage and the Health Treatment Stage Variable

Fixed assets are the capital stock of each country calculated by fixed assets investment in each country by the end of each year. The unit was in billions of dollars.

### Input and Output Variables Statistical Analysis

Table 2 shows a statistical table of the overall input and output variables of old EU states. The minimum values of CO_2_, non-renewable energy, and the mortality rate of children and adults declined from 2010 to 2014. The minimum values of labor, fixed assets, and the survival rate of 65-years-old rose from 2011 to 2014. The minimum values of the renewable energy, GDP, PM2.5, health expenditure, and tuberculosis rate did not change too much.

**Table 2:** Statistics of Input and output variables of old EU member states.

	**Year**	**Labor**	**Fixed assets**	**CO_**2**_**	**Renewable energy**	**Non-renewable energy**	**GDP**	**PM2.5**	**Healthy expenditure**	**Tuberculosis rate**	**Mortality rate of children**	**Mortality rate of Adult**	**Survival rate of 65**
Min.	2010	0.23	0.94	10.97	0.60	15.75	5.32	7.19	0.32	0.00	0.30	11.32	82.48
	2011	0.23	1.15	10.94	0.61	15.72	6.00	7.28	0.30	0.01	0.29	11.24	82.84
	2012	0.24	1.14	10.66	0.66	15.30	5.67	6.60	0.31	0.01	0.28	10.99	83.21
	2013	0.25	1.20	10.05	0.90	14.84	6.17	6.28	0.33	0.00	0.26	10.58	83.64
	2014	0.26	1.32	9.66	1.04	14.06	6.61	6.47	0.35	0.00	0.25	10.27	84.08
Max.	2010	39.09	66.41	758.86	88.92	774.90	341.71	19.06	31.35	0.03	0.52	17.41	88.50
	2011	39.27	76.15	732.50	93.57	727.59	375.77	20.00	33.51	0.06	0.50	16.77	88.75
	2012	39.56	71.28	739.86	99.73	730.31	354.40	18.42	31.72	0.03	0.48	16.15	89.00
	2013	40.00	73.92	757.31	103.05	749.36	375.25	17.78	34.38	0.05	0.46	15.77	89.14
	2014	40.34	77.95	719.88	108.78	704.31	389.87	17.77	36.06	0.02	0.45	15.40	89.29
Ave.	2010	11.69	20.73	199.10	32.88	226.27	104.42	13.17	8.25	0.01	0.40	14.82	85.12
	2011	11.66	22.47	189.54	31.86	211.62	112.62	13.42	8.84	0.01	0.39	14.38	85.47
	2012	11.62	20.72	187.23	34.88	209.44	106.21	12.32	8.38	0.01	0.38	14.02	85.83
	2013	11.60	21.13	183.68	36.93	208.07	110.65	11.98	8.94	0.01	0.38	13.68	86.10
	2014	11.70	21.97	173.14	37.42	195.29	114.47	11.56	9.27	0.01	0.37	13.15	86.38
St.Dev	2010	12.36	21.40	216.63	28.82	237.92	108.87	3.67	9.28	0.01	0.06	1.98	1.94
	2011	12.40	23.77	207.38	27.55	222.08	118.37	3.77	9.89	0.01	0.06	1.81	1.91
	2012	12.47	22.24	209.85	30.53	224.41	112.87	3.49	9.43	0.01	0.06	1.69	1.90
	2013	12.56	22.87	210.74	31.97	226.74	118.07	3.39	10.27	0.01	0.06	1.63	1.83
	2014	12.69	23.97	197.74	32.08	211.67	123.63	3.25	10.84	0.00	0.06	1.56	1.75

*Data Source: World Development Indicators of the World Bank as calculated by authors*.

The maximum values of mortality rate in children and adults declined from 2010 to 2014. The maximum values of the labor, fixed assets, renewable energy, and survival rate of 65-years-old increased from 2010 to 2014. The maximum values of other variables exhibited relatively fluctuating changes from 2010 to 2014.

The average values of the CO_2_, non-renewable energy, PM2.5, and mortality rate of children and adults decreased obviously from 2010 to 2014. The average values of renewable energy, health expenditure, and survival rate of 65-years-old rose from 2011 to 2014. The average values of the labor, fixed assets, GDP, and tuberculosis rate did not change significantly.

Table 3 shows a statistical table of the overall input and output variables of new EU states. The minimum values of PM2.5 and the mortality rate of children and adults declined from 2010 to 2014. The minimum values of labor, renewable energy, non-renewable energy, GDP, health expenditure, and survival rate of 65-years-old rose from 2011 to 2014. The minimum values of fixed assets, CO_2_, and tuberculosis rate did not change significantly.

**Table 3:** Statistics of Input and output variables of new EU member states.

	**Year**	**Labor**	**Fixed assets**	**CO_**2**_**	**Renewable energy**	**Non-renewable energy**	**GDP**	**PM2.5**	**Healthy expenditure**	**Tuberculosis rate**	**Mortality rate of children**	**Mortality rate of adult**	**Survival rate of 65**
Min.	2010	0.16	0.19	2.56	0.02	1.66	0.87	8.52	0.05	0.01	0.32	11.48	61.35
	2011	0.17	0.17	2.54	0.03	1.60	0.95	8.44	0.05	0.00	0.30	1.00	62.25
	2012	0.17	0.17	2.68	0.04	1.66	0.92	7.81	0.05	0.01	0.29	1.00	63.14
	2013	0.18	0.18	2.34	0.05	1.72	1.02	7.48	0.06	0.00	0.27	1.00	63.54
	2014	0.19	0.19	2.35	0.07	1.75	1.13	7.71	0.07	0.00	0.25	1.00	63.93
Max.	2010	16.30	9.72	316.26	25.79	245.98	47.93	27.18	2.20	0.11	1.15	38.23	87.60
	2011	16.36	10.94	317.00	27.64	237.18	52.88	26.37	2.34	0.10	1.13	36.90	87.84
	2012	16.39	9.90	299.93	28.74	234.55	50.04	24.26	2.17	0.09	1.11	35.95	88.09
	2013	16.38	9.86	302.28	29.51	229.02	52.42	22.93	2.36	0.01	1.06	35.74	88.33
	2014	16.74	10.76	285.74	29.03	221.92	54.54	22.21	2.41	0.09	1.00	33.48	88.56
Ave.	2010	3.47	2.30	55.64	8.02	47.57	10.36	18.90	0.51	0.03	0.63	24.71	73.71
	2011	3.45	2.56	55.77	8.00	46.61	11.40	19.02	0.54	0.02	0.60	21.96	74.45
	2012	3.46	2.33	52.74	8.43	45.65	10.66	17.61	0.50	0.03	0.58	21.33	75.19
	2013	3.46	2.37	51.26	8.80	44.33	11.20	16.95	0.54	0.00	0.56	20.68	75.52
	2014	3.53	2.47	49.60	8.78	43.06	11.53	16.40	0.55	0.02	0.53	20.11	75.85
St.Dev	2010	4.52	2.76	84.46	7.78	65.49	12.83	4.45	0.61	0.03	0.26	8.36	8.40
	2011	4.51	3.09	84.59	7.86	63.22	14.13	4.53	0.65	0.03	0.25	10.11	8.13
	2012	4.53	2.79	80.05	8.16	62.64	13.30	4.14	0.60	0.03	0.25	9.75	7.86
	2013	4.53	2.77	80.41	8.39	61.17	13.94	3.92	0.66	0.00	0.24	9.60	7.82
	2014	4.61	2.99	76.09	8.39	59.24	14.47	3.67	0.66	0.02	0.22	9.36	7.78

*Data Source: World Bank open data as calculated by authors*.

The maximum values of renewable energy and survival rate of 65-years-old increased from 2010 to 2014. The maximum values of non-renewable energy and PM2.5 decreased obviously from 2010 to 2014. The maximum values of other variables exhibited a relatively volatile change from 2010 to 2014.

The average values of non-renewable energy and the mortality rate of children and adults decreased obviously from 2010 to 2014. The average values of survival rate of 65-years-old rose from 2011 to 2014. The average values of other variables exhibited a fluctuate change. These variables did not change too much from 2010 to 2014, however.

### Total Annual Efficiency Scores in Meta-Frontier

The overall efficiencies of old EU states from 2010 to 2014 in the meta-frontier are shown in the Table 4. An overall efficiency of 1 in all 4 years was achieved by Germany, Ireland, Luxembourg, and Sweden. The overall efficiency of Denmark, Finland, France, Italy, the Netherlands, and the United Kingdom were near 1, and the efficiency of these countries was never below 0.9 from 2010. Therefore, most of the old EU states' efficiency was over 0.9 in average.

**Table 4:** Overall efficiency by old EU member states from 2010 to 2014 in the meta-frontier.

	**2010**	**2011**	**2012**	**2013**	**2014**	**Annual average**
Austria	0.8161	0.8838	0.8273	0.8248	0.8253	0.8354
Belgium	0.8654	0.9431	0.8751	0.8652	0.8592	0.8816
Denmark	0.9709	1.0000	0.9493	0.9000	0.9430	0.9526
Finland	1.0000	1.0000	0.9853	0.9842	0.9838	0.9906
France	0.9628	0.9987	0.9586	0.9541	0.9499	0.9648
Germany	1.0000	1.0000	1.0000	1.0000	1.0000	1.0000
Greece	1.0000	0.7666	1.0000	0.6185	0.7008	0.8172
Ireland	1.0000	1.0000	1.0000	1.0000	1.0000	1.0000
Italy	1.0000	0.9751	0.9822	0.9569	0.9461	0.9720
Luxembourg	1.0000	1.0000	1.0000	1.0000	1.0000	1.0000
Netherlands	0.9951	0.9786	0.9980	0.9741	1.0000	0.9892
Portugal	0.7677	0.8005	0.7091	0.7269	0.7285	0.7465
Spain	0.9394	0.9547	0.8913	0.9039	0.8658	0.9110
Sweden	1.0000	1.0000	1.0000	1.0000	1.0000	1.0000
United Kingdom	1.0000	1.0000	1.0000	1.0000	0.9794	0.9959
Average of 15 old EU member states	0.9545	0.9534	0.9451	0.9139	0.9188	0.9371

*Data Source: World Bank open data and calculated by authors*.

The other old EU states have a tendency to fluctuate. The efficiency of Austria and Belgium each year was between 0.8 to 0.9 from 2010 to 2014. Greece reached the lowest efficiency, below 0.7, in 2013, but its overall efficiency was over 0.8. Portugal reached the highest efficiency, over 0.8, in 2011, and the overall efficiency was below 0.8. The efficiency of Spain was over 0.8 in total. Thus, the old EU states exhibited a high efficiency—most of the states were over 0.8 in average.

As it shown in the Table 5, there are two states where overall efficiencies in the meta-frontier were 1 in all 5 years in the new EU states. These countries were Estonia and Malta. There were 4 years that Cyprus's efficiency was over 0.9, but it declined below 0.9 in 2014.

**Table 5:** Overall efficiency by new EU member states from 2010 to 2014 in the meta-frontier.

	**2010**	**2011**	**2012**	**2013**	**2014**	**Annual average**
Bulgaria	0.3551	0.9987	0.8600	0.6908	0.7380	0.7285
Croatia	0.3833	0.7298	0.3573	0.3740	0.3737	0.4436
Cyprus	0.9479	0.9114	0.9121	0.9033	0.8762	0.9102
Czech Republic	0.5785	0.9794	0.5754	0.8328	0.5155	0.6963
Estonia	1.0000	1.0000	1.0000	1.0000	1.0000	1.0000
Hungary	0.3661	0.3377	0.3570	0.3310	0.3664	0.3516
Latvia	0.4453	0.6717	0.4925	0.5098	0.4975	0.5234
Lithuania	0.3849	0.6365	0.3916	0.4154	0.3966	0.4450
Malta	1.0000	1.0000	1.0000	1.0000	1.0000	1.0000
Poland	0.3933	0.5429	0.3913	0.3959	0.3804	0.4208
Romania	0.3880	0.3605	0.3404	0.3881	0.3361	0.3626
Slovakia	0.3989	0.3373	0.4575	0.3407	0.3831	0.3835
Slovenia	0.6031	0.7478	0.7473	0.7421	0.7299	0.7140
Average of 13 new EU member states	0.5573	0.7118	0.6063	0.6095	0.5841	0.6138

*Data Source: World Bank open data and calculated by authors*.

Croatia, Hungary, Poland, Romania, and Slovakia 's efficiencies were basically between 0.3 and 0.4. Meanwhile, Croatia's efficiency was over 0.7, and Poland's efficiency was over 0.5 in 2011. Bulgaria, the Czech Republic, and Slovenia's efficiencies were basically over 0.5, though with the exception of Bulgaria's efficiency in 2011. Latvia and Lithuania 's efficiencies were between 0.3 and 0.7, which means their efficiencies have a tendency to fluctuate during the period.

The overall efficiencies in the meta-frontier in new EU states were worse than those in old EU states. There is only one state where its overall efficiency in the meta-frontier was below 0.8 in old Eu states. Compared with new EU states, there were just two states where their overall efficiencies in the meta-frontier were over 0.9. This result can be also be proven by the average value of overall efficiencies in the meta-frontier in old EU and new EU states. The average values of overall efficiencies in the meta-frontier each year from 2010 to 2014 were higher in old EU states than in new EU states.

### Total Annual Efficiency Scores in Group frontier

The overall efficiencies of old EU states from 2010 to 2014 in the group frontier are shown in the Table 6. An overall efficiency of 1 in all 4 years was achieved by Germany, Greece, Ireland, Luxembourg, and Sweden. The overall efficiency of Denmark, Finland, France, Italy, the Netherlands, and the United Kingdom were near 1, and the efficiency of these countries were above 0.9 from 2010. Therefore, most of the old EU states' efficiencies were over 0.9 on average.

**Table 6:** Overall efficiency by old EU member states from 2010 to 2014 in the group frontier.

	**2010**	**2011**	**2012**	**2013**	**2014**	**Annual average**
Austria	0.8561	0.9218	0.8755	0.8849	0.8514	0.8779
Belgium	0.9128	0.9649	0.9175	0.9123	0.8788	0.9173
Denmark	0.9708	1.0000	1.0000	0.9401	0.9477	0.9717
Finland	1.0000	1.0000	0.9852	0.9842	0.9837	0.9906
France	0.9628	1.0000	0.9588	0.9791	0.9505	0.9702
Germany	1.0000	1.0000	1.0000	1.0000	1.0000	1.0000
Greece	1.0000	1.0000	1.0000	1.0000	1.0000	1.0000
Ireland	1.0000	1.0000	1.0000	1.0000	1.0000	1.0000
Italy	1.0000	0.9969	0.9822	0.9759	0.9626	0.9835
Luxembourg	1.0000	1.0000	1.0000	1.0000	1.0000	1.0000
Netherlands	0.9986	0.9970	0.9988	1.0000	1.0000	0.9989
Portugal	0.7677	0.8264	0.7149	0.7469	0.7393	0.7591
Spain	0.9394	0.9547	0.8913	0.9056	0.8658	0.9114
Sweden	1.0000	1.0000	1.0000	1.0000	1.0000	1.0000
United Kingdom	1.0000	1.0000	1.0000	1.0000	0.9807	0.9961
Average of 15 old EU member states	0.9605	0.9774	0.9549	0.9553	0.9440	0.9584

*Data Source: World Bank open data and calculated by authors*.

The other four old EU states have a tendency to fluctuate. The efficiency of Austria, Belgium, and Spain of each year were above 0.8 from 2010 to 2014. Austria reached the highest efficiency, above 0.9, in 2014. Belgium reached the lowest efficiency, below 0.9, in 2014. Meanwhile, Portugal reached the highest efficiency, over 0.8, in 2011, and the overall efficiency was below 0.8. Thus, the old EU states have a high efficiency where almost all of these states were over 0.8 in average.

As shown in the Table 7, in the new EU states, there are six states where overall efficiencies were 1 in all 5 years. These countries are Cyprus, the Czech Republic, Estonia, Malta, Poland, and Slovenia. There are 4 years where Croatia's efficiency was between 0.6 and 0.7, but it reached the highest efficiency, 1, in 2014. There were 4 years that Latvia's efficiency was between 0.7 and 0.9, but it reached the highest efficiency, 1, in 2014.

**Table 7:** Overall efficiency by new EU member states from 2010 to 2014 in the group frontier.

	**2010**	**2011**	**2012**	**2013**	**2014**	**Annual average**
Bulgaria	0.7330	0.9987	0.8600	0.8118	0.7891	0.8385
Croatia	0.6568	1.0000	0.6605	0.6739	0.6662	0.7315
Cyprus	1.0000	1.0000	1.0000	1.0000	1.0000	1.0000
Czech	1.0000	1.0000	1.0000	1.0000	1.0000	1.0000
Estonia	1.0000	1.0000	1.0000	1.0000	1.0000	1.0000
Hungary	0.7375	0.7436	0.7430	0.7184	0.7545	0.7394
Latvia	0.7717	1.0000	0.8590	0.8598	0.8238	0.8629
Lithuania	0.6687	0.9928	0.7334	0.7385	0.7289	0.7725
Malta	1.0000	1.0000	1.0000	1.0000	1.0000	1.0000
Poland	1.0000	1.0000	1.0000	1.0000	1.0000	1.0000
Romania	0.8127	0.9105	0.9207	0.9131	0.8208	0.8756
Slovakia	0.7744	0.6808	0.9279	0.6944	0.7313	0.7618
Slovenia	1.0000	1.0000	1.0000	1.0000	1.0000	1.0000
Average of 13 new EU member states	0.8581	0.9482	0.9003	0.8777	0.8704	0.8909

*Data Source: World Bank open data and calculated by authors*.

Bulgaria and Hungary's efficiencies were basically over 0.7. Meanwhile, Bulgaria's efficiency was over 0.9 in 2011. Lithuania and Slovak's efficiencies were basically over 0.6. Lithuania's efficiency was over 0.9 in 2011, and Slovak's efficiency was over 0.9 in 2012. Romania's efficiencies were between 0.8 and 0.9., and Bulgaria, Croatia, Latvia, Lithuania, and Slovakia exhibited a tendency to fluctuate during this period.

The overall efficiencies in the group frontier in new EU states were worse than those in old EU states. There is only one states where overall efficiency was below 0.8 in old EU states. Compared with new EU states, there were just five states where overall efficiencies in group frontier were over 0.9. We can also prove this from the average value of overall efficiencies in group frontier in old EU and new EU states. The average values of overall efficiencies in the group frontier were higher in old EU states than that in new EU states each year from 2010 to 2014.

### Total Average Efficiency Scores Analysis in Each Stage

From the view of each stage, the overall efficiencies of old EU and new EU states exhibited different performances. We can see the results when we compare Tables 8, 9. In stage 1 (production stage), only the average efficiencies of two old states were below 0.8—Greece and Portugal. When compared with new EU states' efficiencies, there only the average efficiencies of three states were over 0.8— Cyprus, Estonia, and Malta. The lowest old EU states' efficiency in stage 1 was 0.61, which was in Greece in 2014, while the lowest new EU states' efficiency was 0.33, which was in Bulgaria in 2010.

**Table 8:** Average overall efficiency in old EU member states from 2010 to 2014 in each stage.

	**2010 (I)**	**2011 (I)**	**2012 (I)**	**2013 (I)**	**2014 (I)**	**Stage (I)**	**2010 (II)**	**2011 (II)**	**2012 (II)**	**2013 (II)**	**2014 (II)**	**Stage (II)**
Austria	0.85	0.85	0.83	0.83	0.85	0.84	0.78	0.92	0.82	0.82	0.80	0.83
Belgium	0.99	1.00	0.98	0.99	0.96	0.99	0.75	0.89	0.77	0.75	0.76	0.79
Denmark	1.00	1.00	1.00	1.00	1.00	1.00	0.94	1.00	0.90	0.81	0.89	0.91
Finland	1.00	1.00	0.97	0.97	0.97	0.98	1.00	1.00	1.00	1.00	1.00	1.00
France	1.00	1.00	1.00	1.00	1.00	1.00	0.93	1.00	0.92	0.91	0.90	0.93
Germany	1.00	1.00	1.00	1.00	1.00	1.00	1.00	1.00	1.00	1.00	1.00	1.00
Greece	1.00	0.66	1.00	0.67	0.61	0.79	1.00	0.89	1.00	0.56	0.82	0.85
Ireland	1.00	1.00	1.00	1.00	1.00	1.00	1.00	1.00	1.00	1.00	1.00	1.00
Italy	1.00	0.96	0.96	0.92	0.90	0.95	1.00	0.99	1.00	1.00	1.00	1.00
Luxembourg	1.00	1.00	1.00	1.00	1.00	1.00	1.00	1.00	1.00	1.00	1.00	1.00
Netherlands	1.00	1.00	1.00	1.00	1.00	1.00	0.99	0.96	1.00	0.95	1.00	0.98
Portugal	0.74	0.71	0.64	0.67	0.65	0.68	0.79	0.91	0.80	0.79	0.83	0.82
Spain	0.94	0.91	0.81	0.83	0.76	0.85	0.94	1.00	0.98	0.99	0.98	0.98
Sweden	1.00	1.00	1.00	1.00	1.00	1.00	1.00	1.00	1.00	1.00	1.00	1.00
United Kingdom	1.00	1.00	1.00	1.00	1.00	1.00	1.00	1.00	1.00	1.00	0.96	0.99
Average of 15 old EU member states	0.97	0.94	0.95	0.92	0.91	0.94	0.94	0.97	0.95	0.91	0.93	0.94

*Data Source: World Bank open data and calculated by authors*.

**Table 9:** Average overall efficiency in new EU member states from 2010 to 2014 in each stage.

	**2010 (I)**	**2011 (I)**	**2012 (I)**	**2013 (I)**	**2014 (I)**	**Stage (I)**	**2010 (II)**	**2011 (II)**	**2012 (II)**	**2013 (II)**	**2014 (II)**	**Stage (II)**
Bulgaria	0.33	1.00	0.75	0.50	0.56	0.63	0.41	1.00	1.00	1.00	1.00	0.88
Croatia	0.52	0.54	0.44	0.46	0.46	0.48	0.19	1.00	0.22	0.25	0.24	0.38
Cyprus	0.90	0.83	0.83	0.82	0.77	0.83	1.00	1.00	1.00	1.00	1.00	1.00
Czech Republic	0.41	0.96	0.38	0.70	0.34	0.56	0.85	1.00	0.90	1.00	0.81	0.91
Estonia	1.00	1.00	1.00	1.00	1.00	1.00	1.00	1.00	1.00	1.00	1.00	1.00
Hungary	0.42	0.38	0.39	0.39	0.40	0.39	0.29	0.27	0.30	0.23	0.32	0.28
Latvia	0.40	0.47	0.46	0.47	0.47	0.46	0.52	1.00	0.55	0.57	0.53	0.63
Lithuania	0.42	0.46	0.42	0.45	0.44	0.44	0.32	0.92	0.35	0.36	0.33	0.46
Malta	1.00	1.00	1.00	1.00	1.00	1.00	1.00	1.00	1.00	1.00	1.00	1.00
Poland	0.38	0.37	0.37	0.38	0.35	0.37	0.42	0.83	0.42	0.43	0.43	0.51
Romania	0.51	0.42	0.37	0.40	0.39	0.42	0.21	0.26	0.29	0.37	0.25	0.28
Slovakia	0.45	0.42	0.43	0.41	0.43	0.43	0.32	0.22	0.50	0.23	0.32	0.32
Slovenia	0.48	0.57	0.57	0.56	0.54	0.54	0.78	1.00	1.00	1.00	1.00	0.96
Average of 13 new EU member states	0.56	0.65	0.57	0.58	0.55	0.58	0.56	0.81	0.66	0.65	0.63	0.66

*Data Source: World Bank open data and calculated by authors*.

In stage 2, only the average efficiency of one old state was below 0.8—Belgium. When compared with new EU states' efficiencies, there were six average state efficiencies that were over 0.8: Bulgaria, Cyprus, the Czech Republic, Estonia, Malta, and Slovenia. The lowest old EU states' efficiency in stage 2 was 0.56—Greece in 2013—while the lowest new EU states' efficiency was 0.19—Croatia in 2010. We can also arrive at the same conclusion when we compare the Average of 15 old EU member states' efficiency and Average of 13 new EU member states' efficiency in each year.

### The Technical Efficiency of the Group Frontier for Old and New EU Member States

We can learn the technical efficiency of the group frontier for old EU and new EU states from the technology gap ratio (TGR) of EU and non-EU countries from 2010 to 2014, as shown in Table 10. In the old EU states, there were five states where TGRs were 1 in all 5 years. These states were Finland, Germany, Ireland, Luxembourg, and Sweden. There were 4 years where Spain and the United Kingdom's efficiencies were 1 in 5 years. There were 2 years that Denmark, Greece, and Italy's efficiencies were 1 in 5 years. There was 1 year that France and the Netherlands's efficiencies were 1 in 5 years. In the new EU states, there were two states where TGRs were 1 in all 5 years. These states were Estonia and Malta. There were 2 years where Bulgaria's efficiency was 1 in 5 years.

**Table 10:** Average overall TGRs of old EU member states from 2010 to 2014.

	**2010**	**2011**	**2012**	**2013**	**2014**	**Annual average**
Austria	0.9532	0.9587	0.9448	0.9320	0.9692	0.9516
Belgium	0.9480	0.9773	0.9538	0.9484	0.9777	0.9610
Denmark	1.0000	1.0000	0.9493	0.9573	0.9950	0.9803
Finland	1.0000	1.0000	1.0000	1.0000	1.0000	1.0000
France	1.0000	0.9987	0.9998	0.9745	0.9994	0.9945
Germany	1.0000	1.0000	1.0000	1.0000	1.0000	1.0000
Greece	1.0000	0.7666	1.0000	0.6185	0.7008	0.8172
Ireland	1.0000	1.0000	1.0000	1.0000	1.0000	1.0000
Italy	1.0000	0.9781	1.0000	0.9805	0.9828	0.9883
Luxembourg	1.0000	1.0000	1.0000	1.0000	1.0000	1.0000
Netherlands	0.9965	0.9816	0.9992	0.9741	1.0000	0.9903
Portugal	0.9999	0.9686	0.9919	0.9732	0.9853	0.9838
Spain	1.0000	1.0000	1.0000	0.9981	1.0000	0.9996
Sweden	1.0000	1.0000	1.0000	1.0000	1.0000	1.0000
United Kingdom	1.0000	1.0000	1.0000	1.0000	0.9987	0.9997
Average of 15 old EU member states	0.9932	0.9753	0.9893	0.9571	0.9739	0.9778

*Data Source: World Bank open data and calculated by authors*.

Tables 10, 11 shown that the average overall TGRs were obviously higher for each year in old EU states than those states in new EU. When compared with old EU states and new EU states, the average overall TGRs were above 0.95 in old EU states each year from 2010 to 2014, which is higher than those between 0.6 and 0.8 in new EU states.

**Table 11:** Average overall TGRs of new EU member states from 2010 to 2014.

	**2010**	**2011**	**2012**	**2013**	**2014**	**Annual average**
Bulgaria	0.4844	1.0000	1.0000	0.8510	0.9352	0.8541
Croatia	0.5836	0.7298	0.5409	0.5550	0.5608	0.5941
Cyprus	0.9479	0.9114	0.9121	0.9033	0.8762	0.9102
Czech Republic	0.5785	0.9794	0.5754	0.8328	0.5155	0.6963
Estonia	1.0000	1.0000	1.0000	1.0000	1.0000	1.0000
Hungary	0.4964	0.4542	0.4804	0.4607	0.4856	0.4755
Latvia	0.5769	0.6717	0.5734	0.5929	0.6039	0.6038
Lithuania	0.5755	0.6411	0.5339	0.5624	0.5441	0.5714
Malta	1.0000	1.0000	1.0000	1.0000	1.0000	1.0000
Poland	0.3933	0.5429	0.3913	0.3959	0.3804	0.4208
Romania	0.4774	0.3959	0.3697	0.4251	0.4095	0.4155
Slovakia	0.5151	0.4955	0.4930	0.4906	0.5239	0.5036
Slovenia	0.6031	0.7478	0.7473	0.7421	0.7299	0.7140
Average of 13 new EU member states	0.6332	0.7361	0.6629	0.6778	0.6589	0.6738

*Data Source: World Bank open data and calculated by authors*.

### The Efficiency of the Input and Output Variables

We can learn the energy efficiencies for the inputs and outputs from the production stage in old EU states and new EU states. As the Table 12 shows, the GDP efficiencies were above 0.92 each year for old EU states and above 0.68 for new EU states. The non-renewable energy, renewable energy, labor, PM2.5, and CO_2_ efficiencies of old EU states were all higher than the ones of new EU states from 2010 to 2014. The gap of labor, renewable energy, non-renewable energy, and CO_2_ efficiencies between old EU states and new EU states were more significant. There is much more space for the new EU states to improve the energy efficiencies of inputs and outputs. Meanwhile, PM2.5 efficiencies were obviously higher than the CO_2_ efficiencies for old EU states and new EU states.

**Table 12:** Comparison of energy efficiencies of old and new EU member states from 2010 to 2014.

		**Labor**	**GDP**	**Renewable energy**	**Non-renewable energy**	**CO_**2**_**	**PM2.5**
2010	Old	0.9447	0.9828	0.9745	0.9566	0.9394	1.0000
	New	0.6085	0.6952	0.7841	0.7320	0.6625	0.8205
2011	Old	0.9276	0.9673	0.9720	0.9365	0.9064	0.9801
	New	0.6883	0.7216	0.7390	0.7898	0.8370	0.9408
2012	Old	0.9473	0.9711	0.9231	0.9400	0.9346	1.0000
	New	0.6385	0.7050	0.7662	0.7376	0.7404	0.8857
2013	Old	0.9230	0.9838	0.9163	0.9235	0.8867	1.0000
	New	0.6344	0.7204	0.7700	0.7453	0.7792	0.8704
2014	Old	0.9219	0.9529	0.8881	0.9168	0.8771	1.0000
	New	0.6246	0.6855	0.7430	0.7302	0.7371	0.9586

*Data Source: World Bank open data and calculated by authors*.

We can learn the health efficiencies for the inputs and outputs from the health treatment stage in old EU states and new EU states. From the Table 13, we can see the survival rate efficiencies were all above 0.94 for each year in old EU states and new EU states. The health expenditure efficiencies, tuberculosis rate efficiencies, mortality rate of children efficiencies, and mortality rate of adult efficiencies of old EU states were all higher than those of new EU states from 2010 to 2014. Meanwhile, the mortality rate of children efficiencies is lower than the mortality rate of the adult efficiencies for old EU states and new EU states. In conclusion, there is much more space for new EU states to improve their health efficiencies, especially in health expenditure efficiencies, which shows a significant shortage when compared with old EU states.

**Table 13:** Comparison of health efficiencies of old and new EU member states from 2010 to 2014.

		**Health expenditure**	**Tuberculosis rate**	**Mortality rate of children**	**Mortality rate of the adult**	**Survival rate of 65-years-old**
2010	Old	0.8962	0.9828	0.8619	0.9265	0.9720
	New	0.5755	0.6952	0.6954	0.8099	0.9464
2011	Old	0.9365	0.9673	0.9786	0.9846	0.9881
	New	0.8143	0.7216	0.8836	0.9356	0.9730
2012	Old	0.9134	0.9711	0.8899	0.9221	0.9745
	New	0.6680	0.7050	0.7767	0.8353	0.9564
2013	Old	0.8902	0.9838	0.8729	0.9408	0.9634
	New	0.6591	0.7204	0.8333	0.8971	0.9516
2014	Old	0.8783	0.9529	0.8599	0.8945	0.9683
	New	0.6459	0.6855	0.7222	0.7704	0.9422

*Data Source: World Bank open data and calculated by authors*.

The energy efficiencies are very low, especially in terms of the efficiency of CO_2_. Meanwhile, a different performance existed between old EU states and new EU states. Thus, we analyzed the specific situation of energy efficiencies of each old EU states and new EU states in detail. The results are shown in the Table 14.

**Table 14:** Comparison of the annual average energy efficiencies of old and new EU member states.

	**Labor**	**GDP**	**Renewable energy**	**Non-renewable energy**	**CO_**2**_**	**PM2.5**
Austria	0.8901	0.9199	0.9627	0.8342	0.7613	0.9927
Belgium	0.9913	0.9961	0.9896	0.6840	0.7233	0.9926
Denmark	1.0000	1.0000	1.0000	1.0000	1.0000	1.0000
Finland	0.9863	0.9925	0.5014	0.9907	1.0000	1.0000
France	1.0000	1.0036	1.0000	1.0000	1.0000	1.0000
Germany	1.0000	1.0003	1.0000	1.0000	1.0000	1.0000
Greece	0.7976	0.9200	0.8690	0.8753	0.7423	0.9970
Ireland	1.0000	0.9992	1.0000	1.0000	1.0000	1.0000
Italy	0.9694	0.9880	0.9727	0.9596	0.8333	0.9580
Luxembourg	1.0000	0.9944	1.0000	1.0000	1.0000	1.0000
Netherlands	1.0000	1.0062	1.0000	1.0000	1.0000	1.0000
Portugal	0.5032	0.8259	0.8041	0.7622	0.7084	1.0000
Spain	0.8553	0.9413	0.9223	0.9140	0.8642	1.0000
Sweden	1.0000	0.9946	1.0000	1.0000	1.0000	1.0000
United Kingdom	1.0000	0.9914	1.0000	1.0000	1.0000	1.0000
Average of 15 old members	0.9329	0.9716	0.9348	0.9347	0.9088	0.9960
Bulgaria	0.5714	0.6247	0.8412	0.8198	0.8681	0.8874
Croatia	0.5513	0.6527	0.7977	0.6420	0.6497	0.7936
Cyprus	0.7784	0.9424	0.8438	0.9170	1.0000	1.0000
Czech Republic	0.7229	0.6782	0.7251	0.7618	0.6939	0.8760
Estonia	1.0000	0.9844	1.0000	1.0000	1.0000	1.0000
Hungary	0.5576	0.5876	0.6797	0.6525	0.6271	0.8743
Latvia	0.5254	0.5979	0.6611	0.6163	0.7668	0.7825
Lithuania	0.4980	0.6029	0.7588	0.6138	0.6726	0.7997
Malta	1.0000	0.9872	1.0000	1.0000	1.0000	1.0000
Poland	0.4920	0.5794	0.5285	0.6637	0.4180	0.8783
Romania	0.3135	0.5712	0.7681	0.5951	0.4619	1.0000
Slovakia	0.5613	0.6464	0.5688	0.6914	0.6625	0.7845
Slovenia	0.7333	0.7169	0.7130	0.7372	0.9457	0.9616
Average of 13 new members	0.6389	0.7055	0.7604	0.7470	0.7513	0.8952

*Data Source: World Bank open data and calculated by authors*.

In the old EU states, there are eight states where labor efficiency, renewable energy efficiency, and non-renewable energy efficiency were 1 in all 5 years: Denmark, France, Germany, Ireland, Luxembourg, the Netherlands, Sweden, and the United Kingdom. Most of them are high welfare states. There are only four states where labor efficiencies were below 0.9 in the old EU states: Austria, Greece, Portugal, and Spain. The labor efficiency in Portugal was the lowest, and it was 0.5032. There are only three states where renewable energy efficiencies were below 0.9 in the old EU states, and they were Finland, Greece, and Portugal. The renewable energy efficiency in Finland was the lowest, and it was 0.5014. There are only four states where non-renewable energy efficiencies were below 0.9 in the old EU states, and they are Austria, Belgium, Greece, and Portugal. The non-renewable energy efficiency in Belgium was at the lowest, and it was 0.6840.

There are nine states where CO_2_ efficiencies were 1 in all 5 years, and they were Denmark, Finland, France, Germany, Ireland, Luxembourg, the Netherlands, Sweden, and the United Kingdom. The other countries' CO_2_ efficiencies rate efficiencies were above 0.7 in old EU states. There are 11 states where PM2.5 efficiencies were 1 in all 5 years, and they are Denmark, Finland, France, Germany, Ireland, Luxembourg, the Netherlands, Portugal, Spain, Sweden, and the United Kingdom. The other countries' PM2.5 efficiencies rates were above 0.9 in the old EU states.

In the new EU states, there were two states where labor efficiencies, renewable energy efficiencies, and non-renewable energy efficiencies were 1 in all 5 years, and they were Estonia and Malta. There are eight states where labor efficiencies were below 0.6. They were Bulgaria, Croatia, Hungary, Latvia, Lithuania, Poland, Romania, and Slovakia. There were two states where renewable energy efficiencies were below 0.6. They were Poland and Slovakia. Meanwhile, only Romania's renewable energy efficiency was below 0.6.

There were three states where CO_2_ efficiencies were 1 in all 5 years, and they were Cyprus, Estonia, and Lithuania. There were only two states where CO_2_ efficiencies were below 0.6, and they were Poland and Romania. The CO_2_ efficiency in Poland was the lowest, and it was 0.4180.

There were four states where CO_2_ efficiencies were 1 in all 5 years, and they were Cyprus, Estonia, Lithuania, and Romania. All the 13 new EU states' PM2.5 efficiencies were over 0.78. The PM2.5 efficiencies in Latvia was the lowest, and it was 0.7825.

In addition, we also analyzed the different performances regarding health efficiency of old and new EU member states, especially regarding CO_2_ efficiency. We analyzed the specific situation of energy efficiencies of each old EU states and new EU states in detail. The results are shown in the Table 15.

**Table 15:** Comparison of the annual health efficiencies of old and new EU member states.

	**Health Expenditure**	**Tuberculosis rate**	**Mortality rate of children**	**Mortality rate of the adult**	**Survival rate of 65-years-old**
Austria	0.8746	0.9199	0.8386	0.8686	0.9303
Belgium	0.8505	0.9961	0.7256	0.8447	0.8915
Denmark	0.6774	1.0000	0.9184	0.8241	0.9634
Finland	1.0000	0.9925	1.0000	1.0000	1.0000
France	0.6433	1.0000	0.8362	0.9050	0.9268
Germany	1.0000	1.0000	1.0000	1.0000	1.0000
Greece	0.8687	0.9200	0.9488	0.9089	0.9706
Ireland	1.0000	0.9992	1.0000	1.0000	1.0000
Italy	0.8464	0.9880	0.9534	0.9966	0.9980
Luxembourg	1.0000	0.9944	1.0000	1.0000	1.0000
Netherlands	0.9762	1.0000	0.9738	0.9115	0.9854
Portugal	0.8484	0.8259	0.4220	0.8108	0.9586
Spain	0.9687	0.9413	0.8221	0.9697	0.9827
Sweden	1.0000	0.9946	1.0000	1.0000	1.0000
United Kingdom	0.9898	0.9914	0.9437	0.9654	0.9915
Average of 15 old members	0.9029	0.9716	0.8926	0.9337	0.9733
Bulgaria	0.8829	0.6247	0.9085	0.8912	0.9944
Croatia	0.3877	0.6527	0.7547	0.8525	0.9565
Cyprus	1.0000	0.9424	1.0000	1.0000	1.0000
Czech Republic	0.9228	0.6782	0.9404	0.9712	0.9808
Estonia	1.0000	0.9844	1.0000	1.0000	1.0000
Hungary	0.3005	0.5876	0.8684	0.8099	0.8912
Latvia	0.6370	0.5979	0.4349	0.6899	0.9913
Lithuania	0.4629	0.6029	0.4824	0.7211	0.9745
Malta	1.0000	0.9872	1.0000	1.0000	1.0000
Poland	0.5655	0.5794	0.6719	0.8234	0.8224
Romania	0.2940	0.5712	0.4597	0.6505	0.8647
Slovakia	0.3330	0.6464	0.7067	0.6404	0.9279
Slovenia	0.9571	0.7169	0.9387	0.9956	0.9974
Average of 13 new members	0.6726	0.7055	0.7822	0.8497	0.9539

*Data Source: World Bank open data and calculated by authors*.

In the old EU states, there were five states where Health Expenditure efficiency, Mortality rate of children efficiency, and Mortality rate of the adult efficiency were 1 in all 5 years, and they were Finland, Germany, Ireland, Luxembourg, and Sweden. These states are high welfare states. There were only two states where Health Expenditure efficiencies were below 0.8 in the old EU states, and they were Denmark and France. There was only 1 state where the Tuberculosis rate efficiency was below 0.9 in the old EU states, and it was Portugal. The Tuberculosis rate efficiency in Portugal was also the lowest, and it was 0.8259. There were only two states where the Mortality rate of children efficiencies were below 0.8 in the old EU states, and they were Belgium and Portugal. The Mortality rate of children efficiency in Portugal was the lowest, and it was 0.4220. The Mortality rate of the adult efficiencies were all above 0.8 in old UE states. There was only one state where the Survival rate of 65-years-old efficiency was below 0.9, and it was Belgium.

In the new EU states, there were three states where Health Expenditure efficiencies, Mortality rate of children efficiencies, Mortality rate of the adult efficiencies, and Survival rate of 65-years-old efficiencies were 1 in all 5 years, and they were Cyprus, Estonia, and Malta. There are four states where the Health Expenditure efficiencies were below 0.4, and the lowest value was 0.2940 in Romania. Only half of the new EU states' Health Expenditure efficiencies were above 0.8. There were only three states where Tuberculosis rate efficiencies were above 0.9, and they were Cyprus, Estonia, and Malta. The other new EU states' Tuberculosis rate efficiencies where all below 0.8, and the lowest value was 0.5712—Romania. There were three states where Mortality rate of children efficiencies were below 0.5, and they were Latvia, Lithuania, and Romania, and they were 0.4349, 0.4824, and 0.4597, respectively. There were four states where the Mortality rate of adult efficiencies were below 0.8, and they were Latvia, Lithuania, Poland, and Romania. All the new EU states' Survival rate of 65-years-old efficiency were above 0.8, and the lowest value was 0.8224—Poland.

## Conclusions and Implications

This study focused on the energy efficiencies and health efficiencies in 15 old EU states and 13 new EU states from 2010 to 2014 in individual and total stages. We also calculated the efficiencies for the inputs and outputs of the production and health stage in old EU states and new EU states, including the non-renewable energy, renewable energy, PM2.5, CO_2_, labor, GDP, tuberculosis rate, mortality rate of children, mortality rate of adults, health expenditure efficiencies, and survival rate efficiencies. The conclusions from the analysis follow.

1. Average overall efficiencies each year in old EU states were higher than those of new EU states from 2010 to 2014. An overall efficiency of 1 in all 5 years was achieved by the old EU states Germany, Ireland, Luxembourg, and Sweden as well as the new EU states Estonia and Malta.
2. The old EU states whose average overall efficiencies were lower are all located in the eastern or southern regions of Europe, for example, Austria, Greece, and Portugal. The new EU states whose overall efficiencies were lower are all located in eastern Europe, for example, Bulgaria, Poland, the Czech Republic, Romania, Croatia, Latvia, Lithuania, Slovakia, and Slovenia. These new EU members are developing countries either.
3. Old EU states exhibited higher energy efficiency and health efficiency than new EU states. Meanwhile, the average overall efficiencies of old EU states were higher than those of new EU states each year from 2010 to 2014 in the first stage (production stage). Furthermore, the average overall efficiencies of old EU states were higher than those of new EU states each year from 2010 to 2014 in the second stage (health treatment stage) either.
4. There is much more space for new EU states to improve when it comes to health efficiency. For new EU states, the average overall efficiencies in the second stage were all lower than the average overall efficiencies in the first stage each year. Meanwhile, the TGRs of new EU states were much lower than those of old EU states. Thus, the technical efficiency of the group frontier of old EU states was higher than that of new EU states.
5. The renewable energy efficiencies, non-renewable energy efficiencies, PM2.5 efficiencies, and CO_2_ efficiencies in new EU states were all lower than the efficiencies of old EU states. Thus, there is much more space for new EU states to improve their energy efficiencies regarding input and output. Meanwhile, the PM2.5 efficiencies were obviously higher than the CO_2_ efficiencies for old and new EU states.
6. Compared with the health efficiencies of old and new EU states, there is much more space for new EU states to improve the efficiencies of input and output. This is because the old EU states have an obviously advantage regarding efficiencies in all the items each year. When compared with annual energy efficiencies, the old EU states have eight states whose labor efficiencies, renewable energy efficiencies, non-renewable energy efficiencies, CO_2_ efficiencies, and PM2.5 efficiencies were all 1 in each year. But only two new EU states exhibited efficiencies of 1 in all these items each year, and they were Estonia and Malta. Furthermore, when compared with annual health efficiencies, the old EU states have five states whose Health Expenditure efficiencies, Mortality rate of the adult efficiencies, Mortality rate of children efficiencies, and Survival rate of 65-years-old efficiencies were all 1 each year. Only three of the new EU states exhibited efficiencies of 1 in these items each year, and they were Cyprus, Estonia, and Malta.

Based on the above discussions, we put forward the following suggestions for improving the energy efficiency and the health efficiency for the old EU and new EU states.

1. For the new EU states, the energy efficiencies are too low, and the government should thus strengthen their management strategies to reduce air pollution and carbon dioxide emissions. For the old EU states, the capacity of renewable energy has more space to improve, and the government should therefore encourage the development of the usage of renewable energy. In terms of energy use, it is more preferable to use renewable energy, especially clean energy, such as solar energy, wind energy, and so on. Further attention should be given to increasing R&D funding to raise energy transformation.
2. The internal differences are more serious among the new EU states. The international institutional environment can encourage the developing countries with good performance regarding energy efficiency to assist the countries that do not perform well, and this is especially the case for the developing countries.
3. The varying performances in terms of both energy efficiency and health efficiency between old and new EU members are caused by their respective economic situations. Thus, the old EU states should share their techniques and provide financial assistance to those who require it. This will further promote the integration of Europe.
4. New medical technologies should be widely used in medical and health care to improve health efficiency for the new EU states. This is particularly true for eastern EU countries, as they still exhibit a wide gap within which to improve their children's mortality rate.

## Data Availability Statement

The raw data supporting the conclusions of this article will be made available by the authors, without undue reservation, to any qualified researcher. World Bank open data https://data.worldbank.org/. World Bank World Development Indicator https://data.worldbank.org.cn/indicator/.

## Author Contributions

YF and Y-HC: conceptualization. Y-HC: methodology, investigation, and supervision. T-YL: software. YF, Y-HC, and T-YL: validation. XY: formal analysis, resources, data curation, and visualization. YF: writing-original draft preparation and writing-review and editing.

## Conflict of Interest

The authors declare that the research was conducted in the absence of any commercial or financial relationships that could be construed as a potential conflict of interest.
